# Exploring Cultural and Age-Specific Preferences to Develop a Community-Based Colorectal Cancer Screening Intervention for CHamorus and Filipinos in Guam—Findings from a Qualitative Study

**DOI:** 10.3390/ijerph22050746

**Published:** 2025-05-09

**Authors:** Tressa P. Diaz, Santino G. Camacho, Elizabeth J. Elmore, Corinth T. Aguon, Angela Sy

**Affiliations:** 1 Cancer Research Center, University of Guam, Dean’s Circle #7, 303 University Drive, Mangilao, GU 96923, USA; aguonc10838@triton.uog.edu; 2 School of Social Work, University of Washington, 4101 15th Avenue NE, Seattle, WA 98105-6250, USA; sgtino@uw.edu; 3 Margaret Perez Hattori-Uchima School of Health, University of Guam, UOG Station, Mangilao, GU 96923, USA; elmoree@triton.uog.edu; 4 John A. Burns School of Medicine, University of Hawai‘i at Manoa, 651 Ilalo Street, Honolulu, HI 96813, USA; sya@hawaii.edu

**Keywords:** colorectal cancer, cancer disparities, Pacific Islander, cancer screening education, cultural beliefs, Chamorro, Filipino, Guam

## Abstract

The decline in colorectal cancer (CRC) due to screening success in the U.S. is inconsistent across populations and age groups. CHamorus (Chamorros) and Filipinos constitute minorities in the U.S. but comprise over 70% of the population in Guam where steep increases in CRC incidence occur before the age of 50, and only 53.9% of persons have met national screening standards. This preliminary study explored knowledge, cultural beliefs, and age-specific recommendations associated with CRC and screening. Five focus groups segregated by age and gender were conducted with persons aged 40 and above. Data were collected on knowledge, attitudes, beliefs, and screening education recommendations. Focus group participants (*n* = 25) were predominantly CHamoru (60%), Filipino (32%), and female (56%). The mean age was 55. Participants preferred interventions that integrated storytelling from CRC survivors with emphasis on family education rather than limiting to screening-age adults. Multicoders performed an iterative collaborative analysis for the main themes: knowledge of CRC/screening primarily derives from family experiences; increased outreach is needed for men; use of personal narratives; and screening is motivated by family values and intergenerational consciousness. Findings can inform future studies on age- and culturally-tailored early detection strategies to improve CRC screening participation in Pacific populations.

## 1. Introduction

Despite success in colorectal cancer (CRC) screening uptake and declining rates of CRC diagnosis among persons aged 65 and older in the United States (U.S.) [1,2], screening prevalence and reduction in rates are not uniform across all populations. Colorectal cancer diagnoses are rising in adults younger than 50 years of age [2] and CRC screening disparities have been documented in ethnic minorities including Pacific Islander and Filipino populations in the U.S. [3,4] as well as those living in isolated or rural communities [5].

Guam is an unincorporated territory of the United States (U.S.) in the northwestern Pacific Ocean where colorectal cancer (CRC) is the second leading cause of cancer deaths and the third most common cancer on the island [6]. CHamorus (Chamorros) (41%) and Filipinos (35%) are the largest Pacific Islander and Asian groups, respectively, and comprise the majority of the population [7]. Colorectal cancer screening modalities, such as stool-based tests and colonoscopies, are commonly covered by insurance and available at private and public health clinics in Guam but are underutilized. Overall, only 53.9% of all persons aged 50 and above have met U.S. national screening standards compared to 74.3% in the U.S. [8]. Colorectal cancer rates for males are 63% higher in incidence and 43% higher in mortality than females [6]. The majority of CRC cases in Guam are diagnosed at a late stage (44.1%). By ethnicity, late stage diagnosis occurs in 47.7% of cases among CHamorus and 50.0% of cases among Filipinos [6]. CHamorus have higher age-adjusted mortality rates in CRC (23.2) than in the U.S. (14.2) and in the total population in Guam (17.3) [6].

Research on early onset colorectal cancer (EOCRC) among Pacific Islander populations is limited. However, recent studies in Hawai‘i and Guam observed notable disparities for Pacific Islanders in EOCRC incidence compared to Filipinos and other ethnic groups [9,10]. In Guam, relative increases in EOCRC are found in residents ages 35–49, with a significant portion of these cases diagnosed at a late stage [10]. When disaggregated by ethnicity, CHamorus exhibit significantly higher EOCRC incidence rates at younger ages than Filipinos [10].

Regular adherence to CRC screening reduces risk of CRC incidence or death as it facilitates the early detection of precancerous polyps [2]. Cancer screening education interventions led by community health educators (CHEs) are known to improve knowledge and screening uptake in Filipino, CHamoru, and other ethnic minority groups by building community capacity, establishing trust, and implementing evidence-based cancer control interventions [11,12,13,14,15,16]. Often members of the communities they serve, CHEs lead community outreach efforts and provide social support, health education, healthcare system navigation, and linguistic and cultural mediation [11,12,13,14,15,16].

Low screening prevalence, late stage diagnoses, early onset, and high mortality rates necessitate effective early detection interventions to increase chances of survivorship among CHamorus and Filipinos living in Guam. Exploration of age-specific preferences is essential, as U.S. national screening standards have recently lowered the CRC screening eligibility from age 50 to 45 and EOCRC is on the rise, thus making education critical for younger cohorts that may not have considered themselves at risk for CRC. The purpose of this pre-pilot study was to explore CRC screening education preferences of these populations to inform the development of a CHE-led, community-based, and culturally relevant intervention. Aims of the study were (1) to examine cognitive and cultural beliefs associated with CRC and screening among CHamoru and Filipinos and (2) explore recommendations for culturally-tailored and age-specific education interventions.

## 2. Materials and Methods

In this qualitative study, focus groups (FGs) were employed to encourage and facilitate storytelling among participants. Storytelling is a cultural practice among Pacific and Indigenous peoples globally that is safe, familiar, culturally appropriate, and used to facilitate knowledge transmission between generations of Indigenous peoples [17,18,19,20]. A three-member community advisory council composed of a CRC surgeon, community-based cancer organization representative, and CRC survivor guided the study design, implementation, and analyses; a practice drawn from existing community-based participatory research methodologies [21,22]. Each council member was recruited by the Principal Investigator (PI) after an explanation of the purpose and study design.

### 2.1. Participant Recruitment and Eligibility

Participants were recruited for focus groups utilizing purposive and snowball sampling due to the stigma or sensitivity related to CRC screening. Study promotion was facilitated via recruitment fliers that were physically distributed or posted on social media platforms (i.e., Facebook, Instagram, WhatsApp), and by word-of-mouth through existing professional and community networks. These included, but were not limited to, village mayors’ offices, non-profit cancer organizations, private and public health clinics, the University of Guam, and the local cancer control coalition. The study was open to persons who had or had not ever been screened for colorectal cancer, were aged 40 and above, and self-identified as CHamoru and/or Filipino.

### 2.2. Method

Five focus groups were conducted by trained, experienced facilitators online or in-person based on COVID-19 policies and restrictions at the time. Groups were separated by gender to allow increased opportunities for participants to speak freely about their personal experiences, attitudes, and beliefs related to health, colorectal cancer, and medical examinations. Focus group facilitators were matched to the gender of participants to maintain the safety and comfort of participants when sharing their insights.

Focus groups were held over a span of two months. All of the women’s groups were online. When restrictions for public gatherings were lifted, the men’s groups took place in person. Participants were asked a series of questions about their knowledge, attitudes, and beliefs concerning CRC and CRC screening as well as their screening education recommendations (see Appendix A.1). Online or paper polls were utilized within FGs to determine participant preferences and priorities for methods of intervention delivery and characteristics of a community health educator. For in-person FGs, flipcharts were utilized to present polling questions and each FG member was given sticker dots to indicate their choice (see Appendix A.2).

Focus group participants were prompted to identify their preferences for the best setting for themselves and their family to receive messages about CRC screening. They selected their top two preferences among the following options: a family setting (e.g., a CHE could come to speak to their family or attend a family event), a community setting (e.g., at a community center or church), through a self-paced online education session (individual based), and/or from a culturally relevant educational video for public distribution.

Focus groups were also asked what characteristics were important to them when selecting a community health educator for CRC screening. Participants were able to select as many of the following choices that they felt were important to them: (a) was the same gender; (b) the same ethnicity; (c) approximately the same age; and/or (d) could speak the same language (CHamoru or a Filipino dialect) as the participant.

After participants indicated their preferences, an FG facilitator led group discussion about the outcome of the polls and what their rationale was regarding choices. Participants also had the opportunity to talk about options/choices that were not offered in the poll, which they thought were important.

### 2.3. Analysis

Audio recordings of FGs were transcribed verbatim using Descript 49.1.1, a digital transcription software [23]. Data were analyzed using Dedoose 9.0.17, a qualitative software designed for research team use and coding analysis [24]. Two rounds of coding for thematic codes were performed using initial inductive line by line coding and then organized into larger axial code “buckets”. Some examples of these “buckets” include “general knowledge about CRC”, “attitudes and beliefs about CRC screening”, “promotive cultural beliefs and values toward screening”, and “deterrent/restrictive cultural beliefs about screening”.

Collaborative analysis was used to analyze data using inductive dominant thematic coding [25]. During the first round of coding, two team members independently performed line by line coding to familiarize themselves with the data, maintained notes on the data, and generated an initial codebook. The third member of the team reviewed these codes, then the team met to discuss notes and the buckets or categories that would be used in the final codebook for the final round of coding. Team members consulted with one another during coding if they had any questions. After the second round of coding, team members met to discuss emerging themes from the final coded data and codebook. Codes that received the most frequent mentions were prioritized and highlighted as conclusions.

## 3. Results

### 3.1. Participant Demographics

Five focus groups (*n* = 25) were composed of CHamoru (*n* = 15), Filipino (*n* = 8), and mixed CHamoru–Filipino (*n* = 2) men and women aged 40 and above. Among the FGs there were two groups of women aged 50 and above (*n* = 3, *n* = 6), one group of women 40–49 years old (*n* = 5), one group of men aged 50 and above (*n* = 5), and one group of men 40–49 years old (*n* = 6). Additional demographics, including healthcare access and history of CRC screening, are found in Table 1.

### 3.2. Focus Group Themes

Four key themes emerged from the FG interviews: (a) primary source of knowledge about CRC and screening is derived from family experiences; (b) more awareness and outreach needed for men; (c) hearing personal narratives about CRC makes a difference; and (d) screening is motivated by strong family values and an intergenerational consciousness. In our results, participants’ sex and age group are disclosed to contextualize participant attitudes, values, and experiences by age group; however, we do not disclose ethnic background to protect their identity given that the sampling methods and small population in Guam would make participants more easily identifiable.

#### 3.2.1. Primary Source of Knowledge About CRC and Screening Is Derived from Family Experiences

Participants conveyed that transmitted family stories and family lived experiences were their primary source of knowledge about CRC and screening. They relied heavily on family narratives as a source of education in understanding what CRC screening was, what colonoscopy preparation would be like, and what the procedure entailed. These familial stories and experiences created personal and more meaningful lessons on CRC and screening that were internalized by participants.

Focus group members in all groups indicated that they had heard stories from family members and/or friends about CRC or CRC screening experiences, had family that had died from CRC, or had accompanied family to CRC screening or treatment. One participant discussed how she gained a deeper comprehension of the gravity and significance of CRC screening when her nephew was diagnosed and eventually passed away from colorectal cancer.

*“More recently, my knowledge became much more personal when my nephew was diagnosed several years ago at the age of 37 with stage four colorectal cancer. He died two years later…so I learned a lot about the personal impact on an individual, especially a young man and the family through that experience.”*

—Female, aged 50+

This critical time in her nephew’s life was something the participant could not forget and it strengthened her understanding of mechanisms for screening and treatment. Similarly, another participant reported that her knowledge about screening became more definitive when her husband went through the process and that it felt as if she had gone through the screening herself.

*“It’s kind of, we lived through the same experience. I live vicariously through him. And it gave a face to—it became more concrete…more understandable. Now it’s something that I can repeat myself. Right? I can tell others the story. Because there’s a personal touch to it. There’s an emotion attached to it. I understand it more. And I remember it more.”*

—Female, 40–49 years old

These family experiences affected how participants incorporate and heighten their understanding of CRC and the importance of screening. The passing down of stories by family members and shared experiences made these messages consequential and provided salient lessons on CRC screening and prevention.

#### 3.2.2. More Awareness and Outreach Needed for Men

A second theme, especially from the men’s FG, is that much more awareness and outreach is needed for men. This was expressed in terms of men needing to be more open to CRC education and health professionals’ lack of education interventions aimed at men.

Men in the groups, especially those who had not been screened, admitted knowing very little about CRC, screening methods, or preparation for screening. Some were surprised that screening should be routine at the age of 45 and some were not aware that age 50 was the previously recommended screening age. Those who had been screened had been prompted by a physician or had been given information by a family member, which motivated them to get screened.

*“I’m not too familiar with it, but actually, had a friend who died from prostate cancer. But, you know, I know it’s from the same area there (laughs). That’s pretty much what I know about it.”*

—Male, 40–49 years old

Men felt that they were not the focus of cancer education interventions and that more attention was given to campaigns for women, particularly around breast cancer. In the following quotes, participants reflect on how women and men know how vital breast cancer screening is to women’s health due to successful and publicized screening promotion. In the first quote below, the participant acknowledges that men tend to evade medical attention but also suggests that lack of education reduces opportunities for early cancer detection.

*“Now we’re having all sorts of society evolving around that to make sure that we watch women as they go through. Men seem to stay away from that space. ‘If I’m fine, I’m good,’ until a moment in time when you actually have to go in. And then you find out that two years ago, or last year that could have been picked [up].”*

—Male, aged 50+

*“We always hear breast cancer awareness. What about men? I mean, no offense to the female aspect. But like I know more about breasts than that what’s going on with us…... My sisters, they share it, you know, “We’re gonna go get our mammograms,” I-I never said I’m gonna go get, “you know”, to my family. I think the emphasis is not more with the men, but with the women…maybe more awareness with us, you know?”*

—Male, 40–49 years old

The sentiment of needing more outreach for men was not limited to the men’s focus groups. One woman shared a story about how her husband took the initiative himself to ask the physician for screening once he was age eligible and was surprised that it was not offered by the doctor as a routine screening. The idea that physicians have not mentioned CRC screening at medical appointments was also shared in some of the men’s groups.

*“My husband, who’s 65, he had to ask for his colonoscopy. It was not something that his primary physician who—my husband’s very happy with him, but my husband just underwent a colonoscopy, two weeks ago. I asked him, I said, “Did your doctor bring it up or did you have to ask for it?” And he said, “I had to ask. It wasn’t brought up.” And so I do think that there’s perhaps a gender difference in terms of who gets routinely asked or routinely educated and who doesn’t.”*

—Female, aged 50+

*“Well…I mean, I’m 48. My doc, I don’t think that my doctor’s ever brought that up one time with me that maybe I should get screened.”*

—Male, 40–49 years old

Discussions on limited information about CRC and screening led to that on men’s avoidance of medical attention, fear, and not wanting to know about a diagnosis. Male participants acknowledged that they were not likely to seek medical care in general unless symptoms were severe and intensely painful because they were taught that “you only go to the doctor if you’re dying”. They felt there was a prevailing expectation that they be strong and “hold it in”. This was referenced in the second quote below as part of CHamoru cultural expectations of men to be “tough” and “hard”.

*“Just speaking on behalf of my uncle, who’s [sic] never went to the doctor. Never and then just recently, he almost died. He was like, ‘No, I’m still okay. And he went to the doctor and they found out that, you know, he was bleeding inside…and for the years that he’s never been to the doctor and we always ask him why you don’t want to go to the doctor. He said, just like that, “I’m a guy, I don’t need to. I’m okay. I’m okay.” But inside, you know, you’re really hurting. But they just suck it in and drive on, you know?”*

—Male, 40–49 years old

*“There’s the modern-day Chamorro culture that—I grew up in where, you know, you have to be hard, you know, you have to pro-project this image of, you know, a tough guy image and sometimes—That works towards your detriment.”*

—Male, 40–49 years old

Several participants also spoke specifically of the fear of finding out about a cancer diagnosis and the perception that seeking medical attention invites bad news.

*“Other people have this kind of false equivocation with going to the doctor where, ‘Well, I know people who are healthy until they went to the doctor and then…’ And so it might be…a fear of what the doctor will tell you.”*

—Male, 40–49 years old

*“Some people I know do not want to know. Of what they might find. If they do the colonoscopy. It’s like that in itself is daunting, outta sight, out of mind type mentality. And yet I think that, that, continues to let colorectal cancer be the second leading cancer among men.”*

—Male, aged 50+

Even though participants expressed fear of a cancer diagnosis or patterns of delaying medical attention, they also emphasized that they want to be educated by their healthcare provider so that they can make an informed decision as to whether to get screened or not. They suggested more community outreach, a media blitz, public campaigns, and prior notifications for screening eligibility.

*“The doctor should be telling us, almost like a two minute warning. It’s like, ‘Hey man, you know, you’re how old, okay. In two years, you know don’t forget, you’re gonna have to do this n-next year.’ ‘Hey, it’s you know, maybe next year you might want to consider doing this.’ And then the year happens like, ‘Okay, so, this year, you know, you should be doing this.’”*

—Male, 40–49 years old

*“I would like for my doctor, any, even if it’s, based on the numbers of people that are contracting it or acquiring it or whatever the case may be on Guam…if I fit that demographic, can you please bring it up to me? You please let me know.”*

—Male, 40–49 years old

#### 3.2.3. Hearing Personal Narratives About CRC Makes a Difference

The third theme is the power and potency of personal narratives in facilitating CRC education and screening behavior. Focus group members spoke about the importance of hearing personal narratives from those who have survived CRC or who have gone through screening. They noted that it opened a pathway to discussing the typically stigmatized topic of CRC and colonoscopies that involve uneasy conversations about colorectal anatomy.

*“When you’re talking about colorectal cancer, it’s—you’re talking about a very uncomfortable part of one’s body image. We don’t have a comfortable language to talk about it. So being able to hear other people’s stories, it sort of melts the ice and it creates a comfort and sense of “Hey, it’s, it’s okay.” Okayness to talk about something which in our cultures we’re raised to think of as being very, very private, anything related to elimination or sexuality is either very personal and private or it’s made fun of, made jokes about.”*

—Female, aged 50+

Participants said that hearing personal narratives makes a difference because it creates or strengthens a distinct connection between storyteller and listener and makes it more relatable. For participants, the personal aspect of it facilitates learning that engages an emotional link to the information more than formal instruction would. Personal narratives about CRC and the experience of going through CRC screening made it more compelling.

*“I’m especially big on, that you’d want to develop more storytellers, except the story is real. They would not be telling some make believe thing but, their personal accounts or people’s personal accounts of, surviving or in some cases, people, their loved one’s not surviving, you know? And that you don’t need a degree. You don’t need extensive training. You need to just really have the passion that will move people. Cause that’s the biggest thing. I think the personal, the personal connection that you make that will move people.”*

—Male, aged 50+

Hearing personal narratives was not limited to family members in order to be influential. Other interpersonal relationships also served as a means to share CRC screening experiences within community. One participant that had been screened talked about persuading his co-workers to be screened by sharing the story of his colonoscopy experience, highlighting its importance in prevention, and ultimately encouraged them to be screened.

*“When I had coworkers who were older than I was, who never—and whenever we talk about this they’re so ‘No, I would never do this.’ So adamant. ‘I would never do this. They’re never gonna do that to me. It’s not manly for them (light laughter in the group) to do this to me.’ And I was like, ‘Yeah, until you get that thing, and then you find out that you’re going to die.’ I got both of them- my coworkers to go do it. And one of them had to have numerous polyps removed! And I remember him coming back to me and saying, ‘You know what, thank you for that really, real life talk about the, consequences- if we don’t get checked.’”*

—Male, aged 50+

#### 3.2.4. Screening Is Motivated by Strong Family Values and an Intergenerational Consciousness

The last theme is that participants put strong emphasis on the value of family in health decisions and an intergenerational consciousness as primary reasons to get screened for colorectal cancer. Although FG questions included prompts about family, participants spoke repeatedly about immediate and extended family members with regard to the decision to get screened or not.

*“Because like my mom died of cancer. And when we found that she had cancer, we were there with her as a unit, as a family, the moment that she found out until the day she died, you know? So I think it’s best for my family. We did it as a family unit. So if let’s say I found out that I do have it, then at least my family unit—now we need to kick on the bucket and start learning what’s going on, what needs to be done.”*

—Male, 40–49 years old

*“So in my family, how we talk about health issues is, um, looking again at how we value our family…But the conversation also begins with, how old are your children or, oh your daughter is gonna get married. You know, we’d like you to stay longer. We’d like you to be with us. And when you’re with us, we’d like you to be healthy. So if this is something that we need to do and no matter how inconvenient and unpleasant it is, but- if it means we’re gonna enjoy the family more because you’ll be here longer and you’ll be healthy with a good quantity of life, then it’s something that we have to do. And so we always go back to not just ourselves, but who are going to be affected by the choices that we make, by the health choices that we make now. And so that’s how we deal with it with our family.”*

—Female, aged 50+

Being “alive” or present for each other as an act of care and love for family was a key value that some participants felt would be valuable in encouraging people to screen for CRC. Caring for one’s health through screening and preventing cancer related death was viewed as an act of care and love for one another. One participant stated that screening is one of the most loving things you can do for your family.

*“I think an important message to get across because of the family focus is to emphasize love. And what I mean by that is to get across the message, that screening is one of the most loving things you can do for your family. You know, they want you to be around, you wanna be around for your family…I remember when I began using that messaging in my own family and at first, you know, they would laugh at me and think that was sort of funny. But now we talk about it as sort of something, we kind of remind each other, taking care of yourself, screening is about taking care of yourself and it’s the most loving thing you can do for your family.”*

—Female, aged 50+

Intergenerational consciousness is the participants’ keen attention to generations past, present, and future. As a theme, it is associated with expressed family genealogies of cancer (oral histories of family members that survived or died from cancer), accounts of how family members experienced cancer or how they opted for or against treatment/cancer screening, and stories about the passing down of knowledge and values regarding wellness or disease to the next generation.

*“I think the incentive, for everyone is—it’s true that we tend to not want to confront things or put things off. But the incentive is to think about your family or your spouse or your friends, or the people that rely on you that who would not be there. If you have co—polyps and you never have it checked. Because the impact to them is tremendous. I mean, you would, you wanna be there for them. For many, many, many reasons. And I think that’s a message that we can all promote in all kinds of different ways.”*

—Male, aged 50+

*“I still think that family is still highly valued. You know, so if my mom back then were kind of resisting to getting screened [sic]. It’d be like, well, don’t, you wanna see me graduate, address your grandchildren, you know, grow up and get married…It like not just what impacts you. But how it impacts your other family, whether it’s your spouse or your children, you wanna be around for the long haul. And knowing that if you can get screened early and- they can, you know, again, get screened early and deal with the issue then? Then you have a better chance of, I guess, you know, meeting that goal.”*

—Female, aged 50+

The value of family expressed through love, care, and responsibility were central to these FG discussions. Woven through participants’ responses on whether to get screened or not were anecdotes about mothers, fathers, grandparents, spouses, siblings, nieces, nephews, children, and grandchildren. Participants’ concept of family extended beyond the family nucleus and invoked prior and future generations.

### 3.3. Focus Group Poll Results

Polls during the groups gave FG members the opportunity to choose their top preferences with respect to community health educator characteristics and methods of intervention delivery before being influenced by the group discussion.

#### 3.3.1. Community Health Educator (CHE) Characteristics

Based on the polls, all FGs chose learning from a CHE who is similar in age and gender, concordant withs the top two important characteristics. Men’s and women’s FGs explained their preference for learning from someone of the same gender as being more comfortable, having a shared experience, and because women’s and men’s bodies are different. Men’s FGs in particular described how discussing CRC and screening with a male CHE would help increase their identification with related issues. They used phrases such as “a man’s perspective” and “shared connectedness” and that it would be more believable for them that a man would understand their feelings about having a colonoscopy. One participant specified that he was unlikely to discuss any fears he might have if the CHE was a woman.

Learning from a CHE who is similar in age was the second most prioritized characteristic across focus groups. Participants communicated that they would be more at ease with someone that could relate to their age-specific health challenges, that it would be easier to identify with the CHE, and that there would be more credibility from their perspective.

Poll options on CHE characteristics that were not as highly ranked by FG in the study were that the CHE be the same ethnicity as the participant or speak CHamoru or a Filipino dialect. Focus groups aged 50 and above ranked ethnicity higher than the groups of participants in their 40s. Language access was only a priority for women’s focus groups. Participants in those groups indicated that language access might be a factor for other people and possibly older generations. They noted that, even if a person speaks English as their second language, their comprehension would likely be strengthened if the CHE spoke in the person’s primary language. One participant mentioned that she selected her healthcare worker based on their ability to speak the same language as her.

#### 3.3.2. Preferred Methods of Intervention Delivery

Choices for preferred methods of intervention delivery included a community setting, a family setting, a self-paced individual online module, or a culturally tailored video. The top selection across FG was that screening education interventions be conducted in a family setting (64.0%).

A self-paced individual online module and a culturally relevant video were equally represented (52.0%) as a second preferred option across FG, although focus groups of participants in their 40s had less preference for an online module (45.5%) than a culturally-tailored video (63.6%). Conversely, FG of participants aged 50 and above favored an online module (57.1%) over a culturally-tailored video (42.9%).

The opportunity to contribute additional thoughts and suggestions was presented during FG polling discussions. Participants placed heavy emphasis on the importance of storytelling as a method of intervention delivery and highly encouraged interventions aimed at educating families as opposed to screening-eligible participants.

## 4. Discussion

This qualitative study explored CRC knowledge and cultural beliefs of CHamorus and Filipinos residing in Guam, and preferences for CRC screening education interventions that are culturally appropriate and specific to persons 40–49 years, and ages 50 and above in these populations. Study participants expressed that screening is generally motivated by family values and an intergenerational consciousness embodied through love, care, and responsibility for one’s family by tending to and maintaining one’s health. They communicated that more awareness and outreach for CRC screening was needed, primarily for men. There was strong emphasis on educating families rather than focusing solely on screening-eligible individuals for CRC education. Lastly, while participants preferred culturally relevant videos and online modules as methods of delivery, they underline the use of storytelling as an influential and relatable teaching practice.

Study results illuminate the centrality of family and family values in CRC screening education. Participants contextualized their understanding of CRC and screening with stories about their families, wherein families and values tied to families held important roles in learning and encouraging them to engage in CRC screening. Love, care, and responsibility within families and for future generations were viewed as motivating values for CRC screening. Values in CHamoru culture such as inágofli’e’ and inafa’maolek could be essential to CRC screening education. Inágofli’e’ is the virtue of loving one another through interdependent care and by “seeing” or validating each other’s experiences [26]. Inafa’maolek is to restore what has been disrupted and to create collective good through reciprocity [27]. In Filipino culture, the value of kapwa or the recognition of a shared identity and interconnectedness with others may play a role in screening education [28]. These cultural values conceivably underscore the importance of maintaining health to fulfill one’s cultural responsibility to love and care for one’s family and community.

Participants also indicated that more education is necessary to increase public awareness of CRC risk and screening, especially for men. Some noted that healthcare providers did not discuss CRC screening with men. Failure of healthcare providers to recommend CRC screening is a barrier reported in some studies pertaining to men in minority communities [29,30,31]. Lack of knowledge coupled with cultural gender norms related to masculine strength and fear of diagnosis can create multiple barriers to CRC screening participation. Cultural gender norms that include hypermasculine warrior prototypes among CHamoru [32], Pacific Islander [33], and other local men on Guam may underlie the need to maintain an image of “strength” that deteriorates when someone expresses they are feeling sick. Internalized homophobia may also impede men from accessing CRC screening. Previous studies have found that internalized homophobia among cisgender and heterosexual men in minority populations can deter them from engaging in colonoscopies [31,34,35]. Continuous efforts from healthcare providers to provide reminders and screening information, particularly to men, was recommended by participants to address these screening barriers.

When polled about preferred characteristics of a community health educator, participants felt most comfortable learning from a CHE that identified as the same gender. Culturally among CHamoru and Filipino communities, gendered spaces can facilitate greater safety and comfort in knowledge sharing. Colorectal cancer and related screening involves parts of the body that may be considered private and/or sexualized and perceived as a sensitive or taboo conversation topic across gendered groups. Current studies and historical examples suggest that CHamoru women experience mamahlao—a cultural norm for respectful code of conduct, modesty, and shame—related to gynecological health and wellness [36,37], and that this may act as a barrier to cancer screening [36]. Similarly, studies show that discomfort with mammograms or pap tests among Filipino and other Asian women are obstacles to breast and cervical cancer screening uptake, especially for those unwilling to be touched or to expose their bodies for these medical procedures [38,39]. Our study findings on the intersections of health behavior, gender, and culture are consistent with previous research in the Pacific [33,40] and encourage attention to cultural gender norms in cancer screening education.

Mixed delivery for community health educator interventions were highlighted in FG discussion. By age, FGs of participants in their 40s preferred a culturally relevant video and FGs of participants in their 50s preferred an online module as tools for delivery. All FGs regardless of age group stipulated that storytelling be embedded in any type of CRC screening education interventions intended for their communities. Research with Indigenous and minority populations shows that use of narratives and storytelling has been effective in cancer education, health promotion, and community-based health interventions [41,42,43,44,45]. For instance, in Australia, the Aboriginal practice of yarning circles–an oral tradition of sharing stories and transference of knowledge—has been utilized in Aboriginal and Torres Strait communities as Indigenous research methodology and to promote cancer education [41,42]. Furthermore, efforts to incorporate storytelling in culturally appropriate videos in minority communities have contributed to increase in cancer knowledge and intention to get screened [44,45,46]. Digital storytelling is a developing health promotion method in the U.S. that incorporates a storyteller’s images, voice, and music in a short video to convey an event or situation that impacted the person’s life [45]. Studies in Latinx communities have found success in digital stories as a tool for community health educators and in community-based research on cancer education [44,45,46]. One example includes use of a 3- to 5-min digital story on cervical cancer and HPV co-testing composed of photos from an illustrated Spanish-language booklet and voices from a scripted conversation between the characters in the photos that saw significant increases in knowledge about cervical cancer, screening, and HPV risk among Latinx women [45]. Another study partnered with a Latinx church community church to develop and collect feedback from focus groups on digital stories of 2- to 3-min testimonies from five men and five women about colorectal cancer screening [44]. Participants highlighted the powerful impact of hearing personal stories from within their community and felt motivated to complete or continue their CRC screening. Further research on whether digital storytelling leads to significant increases in cancer screening rates in minority communities is needed.

Lastly, participants highly encouraged efforts to educate families rather than limiting education interventions to individual screening-age adults. This is a unique recommendation and, if tested, could lead to a novel approach, as evidence-based colorectal cancer screening interventions in the U.S. tend to target individuals even when considering family history of CRC. Studies using family units as the focus of intervention are limited [47]. One qualitative study modified a family-based behavioral intervention on melanoma for use with families of persons diagnosed with colorectal cancer [47]. The colorectal cancer patients were interviewed for feedback on the intervention materials. However, the full effects of the intervention on families and screening uptake has yet to be tested. Additionally, a randomized control trial recruited individuals with a first degree-relative or multiple family members with CRC for a telephone-based intervention to increase colonoscopy screening [48]. While findings demonstrated an increase in screening adherence, the intervention was aimed at the individual and not the family as a whole.

Collectively, our study findings suggest that community health educators employ strategies with families focused on utilizing stories told by CRC survivors and persons who have been screened, highlighting the value of family and empathetic to intergenerational consciousness, and an awareness of age cohorts and cultural gender norms.

### Limitations

Limitations to the study are that use of non-probability sampling methods and small sample size biases the sample and limits generalizability. Snowball sampling is useful in recruitment for research on stigmatized or sensitive subject matter but potentiates a homogenous sample, as participants may refer individuals similar to themselves. Findings may be applicable only to these ethnic groups in Guam. However, these sampling methods are advantageous to identifying groups of interest, gathering in-depth data from participants, and highlighting their common experiences with CRC and screening, which at its core aligns with the spirit of qualitative research. Another limitation is that collected data was not shared with study participants to verify resonance of the analysis with their experiences. Notwithstanding, steps to assure rigor in the qualitative methodology were taken through use of researcher triangulation and an advisory board to help strengthen the credibility of the data. Coding was performed independently by two research team members (E. E., S.G.C.) and reviewed and corroborated or resolved by a third (T.P.D.) in team discussions. The advisory board supported the analyses and design of our data collection processes, allowing us to confirm data credibility and interpretations of data with both experts and community members [49,50].

## 5. Conclusions

These initial in-depth research findings provide insight into contextual factors that influence CHamoru and Filipino participation in CRC screening and education. Findings may be utilized to develop studies on an age-specific and culturally-tailored CRC screening education intervention facilitated by CHEs for CHamoru and Filipino families to improve CRC screening participation in these underserved minority populations. Although findings are precursory to further research, they may serve to support existing health policies and cancer programs in Guam grounded in family-focused, community-engaged cancer education and prevention. Future research will involve developing and testing a pilot community-based CRC screening promotion based on results from this study.

## Figures and Tables

**Table 1:** Focus group participant demographics.

Variable	Participants (*n* = 25)	
	*n*	%
Sex		
Women	14	56
Men	11	44
Age		
40–49	11	44
50 and above	14	56
Ethnicity		
CHamoru	15	60
Filipino	8	32
Mixed CHamoru/Filipino	2	1
Healthcare Access		
Has regular clinic	23	92
Has regular provider	21	84
Has health insurance	24	96
CRC Screening		
Ever had a FIT/FOBT	6	24
Ever had a colonoscopy	14	56

## Data Availability

Data presented in this study is unavailable due to issues of ethics and privacy. This is to protect the confidentiality of participants as the study sampling methods and small population in Guam could make participant narratives identifiable.

## Abbreviations

The following abbreviations are used in this manuscript:

CHE	Community Health Educator
CRC	Colorectal Cancer
EOCRC	Early Onset Colorectal Cancer
FG	Focus Groups
U.S.	United States of America

## Appendix A

### Appendix A.1. Interview Guide Questions

**Initial Question**	**Follow-Up Probes**
1. Tell us what you know about colorectal cancer (CRC)?	- What concerns do you have about CRC? - If you feel comfortable sharing, do you have any experiences yourself with CRC or with your family or friends? - What, if any, particular health or cultural beliefs do you have about colorectal cancer?
2. Tell us what you know about CRC screening.	- Who, if anyone, has discussed CRC screening with you? - What does your family think about CRC screening? - What experiences have you and/or your family or friends had with CRC screening?
3. We plan to train community health educators to talk to people about colorectal cancer and screening.	- Who would you feel comfortable with? Who would you listen to? - How important is it that the CHE be the same gender as you? - Be the same ethnicity? - Be around the same age? - Be able to speak and understand your (Filipino) dialect/CHamoru?
4. How would you like to hear about CRC prevention?	- What’s the best setting for you and your family to receive messages about CRC screening? - In a family setting (a CHE could come and speak to your family or attend a family event)? - In a community setting (e.g., at a community center or central location)? - Self-paced online education (individual-based)? - A culturally relevant education video for public distribution? - Others? - Of these delivery methods, which would you prefer? - How useful is it to hear stories of CRC survivors or those who get screened? - What impact, if any, do stories of survivors have in learning about CRC? - How do family and friends communicate what they know about CRC to you? - What are your thoughts on whether an educational video where you, your friends, and family could relate to the culture would be useful to learn about CRC prevention?
5. Think about Chamorro/Filipino traditions and values. What messages would help educate families about colorectal cancer and screening?	- What messages would you and your family and friends like to hear about CRC and screening? - What values do you and your family have around health and screening? - What practices do you and your family have around health and screening?
